# Reply to the letter to the editor regarding “diagnosis, treatment, and outcome prediction of non-convulsive status epilepticus in unconscious patients in intensive care units”

**DOI:** 10.1186/s42466-026-00511-6

**Published:** 2026-07-14

**Authors:** Laurent M. Willems, Adam Strzelczyk

**Affiliations:** Epilepsy Center Frankfurt Rhine-Main, Department of Neurology, Goethe-University and University Medical Center Frankfurt, Theodor-Stern-Kai 7 (Haus 23D), 60596 Frankfurt am Main, Germany

## Dear Editor

we thank León-Ruiz, Castañeda-Cabrero, and Benito-León for their kind interest in our review on non-convulsive status epilepticus (NCSE) [1] and for the thoughtful comments with which they extend the discussion to NCSE in the paediatric population.

Our review was, by design, confined to adult patients with disorders of consciousness on neurological and interdisciplinary intensive care units [2–4]. We deliberately did not address the paediatric setting [5, 6], which differs in several important respects, especially a higher proportion of genetic and idiopathic etiologies, a distinct semiology, age-specific scoring and dosing considerations, and separate diagnostic and therapeutic pathways. We therefore welcome the correspondents’ emphasis on this group, to which they have themselves previously drawn valuable attention [7], as the particular challenges of recognising NCSE in children merit dedicated attention rather than a cursory mention within an adult-focused review.

We fully share the core message the authors highlight. In both adults and children, NCSE is a time-critical diagnosis whose subtle, often purely encephalopathic presentation delays recognition and may worsen outcome regarding morbidity and mortality [1, 8]. Early identification, prompt treatment, and close monitoring are decisive for reducing morbidity and mortality across the entire age spectrum and different etiologies [9–14].

We also concur on the promise of point-of-care EEG (POC-EEG). In our review we discussed POC-EEG as a means of accelerating diagnosis and of offering economic benefit by avoiding unnecessary transfers and shortening hospital stays, while noting that its use remains largely confined to specialised centres [1]. The paediatric data cited by the correspondents [15], in which bedside two-channel POC-EEG enabled earlier detection of NCSE in a paediatric emergency department, provide a welcome illustration of this potential. They equally underline the limited availability of expertise in continuous and quantitative EEG in adult and paediatric critical care alike. In addition, inadequate reimbursement remains a shared obstacle while status epilepticus is associated with high costs [16].

Finally, we agree that further and larger studies are needed to clarify the pathophysiology, epidemiology, and risk factors of NCSE and to develop effective, age-appropriate therapeutic strategies in both populations. We are grateful to the correspondents for helping to raise awareness of NCSE in children and adolescents, and are pleased that our review may serve as a starting point for this important discussion.

## Data Availability

No datasets were generated or analysed during the current study.
